# Genetically engineered FGF1-sericin hydrogel material treats intrauterine adhesion and restores fertility in rat

**DOI:** 10.1093/rb/rbac016

**Published:** 2022-03-09

**Authors:** Chun-Yi Guan, Feng Wang, Lu Zhang, Xue-Cheng Sun, Dan Zhang, Hu Wang, Hong-Fei Xia, Qing-You Xia, Xu Ma

**Affiliations:** 1 Reproductive and Genetic Center of National Research Institute for Family Planning, Beijing 100081, People’s Republic of China; 2 Graduate School, Peking Union Medical College, Beijing 100005, People’s Republic of China; 3 Biological Science Research Center, Chongqing Key Laboratory of Sericultural Science, Chongqing Engineering and Technology Research Center for Novel Silk Materials, Southwest University, Chongqing 400715, People’s Republic of China

**Keywords:** intrauterine adhesions, FGF1-sericin hydrogel, fibrosis, TGF-β/Smad

## Abstract

Endometrial injury can cause intrauterine adhesions (IUA) and induce the formation of endometrial fibrosis, leading to infertility and miscarriage. At present, there is no effective treatment method for severe IUA and uterine basal injury with adhesion area larger than one-third of the uterus. In this study, we prepared FGF1 silk sericin hydrogel material (FGF1-SS hydrogel) to treat endometrial injury and prevent endometrial fibrosis. Compared with the silk sericin hydrogel material (WT-SS hydrogel), FGF1-SS hydrogel significantly promotes the cell migration and infiltration ability of endometrial stromal cells (ESCs). More importantly, FGF1-SS hydrogel can release FGF1 stably for a long time and inhibit the ESCs injury model forms fibrosis through the TGF-β/Smad pathway. In the IUA rat model, FGF1-SS hydrogel treatment effectively restored the number of uterine glands and uterine wall thickness in rats, with a fertility rate of 65.1% ± 6.4%. The results show that FGF1-SS hydrogel is expected to be a candidate to prevent IUA.

## Introduction

The endometrium is a highly active tissue, divided into outer functional layer and inner basal layer according to function [1]. The functional layer falls off every menstrual cycle, and the basal stromal cells differentiate and regenerate to form the epithelium on the surface of the endometrium [2]. Uterine adhesions are caused by uterine surgery to damage the basal layer of the endometrium, which leads to the repair of the basal layer of the endometrium, endometrial fibrosis occurs and the functional layer and the basal layer cannot be distinguished [3]. As a result, the receptivity of the endometrium is decreased, and the uterine cavity and/or cervical canal are partially or completely occluded [4], leading to amenorrhea, secondary infertility and other diseases [5–7]. At present, the most effective method for the treatment of intrauterine adhesions (IUA) is transcervical resection of adhesion, but the postoperative re-adhesion formation rate is high [8]. Yu *et al.* reported that with severe IUA, the postoperative re-adhesion rate was as high as 65% [9]. Hooker *et al.* [10] systematic evaluation showed that the incidence of moderate and severe IUA was 48% when pregnancy was terminated in the first trimester. Therefore, how to effectively prevent postoperative uterine cavity re-adhesion is a difficult point in IUA treatment. Although there are current treatment methods, such as estrogen therapy [11], intrauterine device [12] and sodium hyaluronate [13], the therapeutic effect is not obvious in moderate to severe IUA, and it does not significantly increase the pregnancy rate.

The main pathological feature of IUA is endometrial fibrosis. Therefore, the main idea of treating IUA is to promote endometrial regeneration and inhibit endometrial fibrosis. The physical barrier effect of biological scaffold materials shows a good prospect in the treatment of endometrial injury [14]. Therefore, exploring the treatment options and drug delivery strategies for preventing IUA and finding safer and effective treatment methods have become the object of attention [15]. Sericin is a polymer biomaterial with excellent biocompatibility and is mainly used as a wound dressing [16–18]. Zhang *et al.* [19] found that sericin hydrogel combined with stem cell factors can effectively generate blood vessels and promote collagen deposition. The crosslinking of the porosity of sericin materials can encapsulate growth factors for drug delivery [20–22]. The limited fluidity of the crosslinking of the porosity of the sericin material can encapsulate growth factors and become a functionalized hydrogel with slow-release effect [23–25]. FGF1 factor plays a role in the process of angiogenesis and wound healing, but its short half-life and low concentration at the injured site limit the use of FGF1 [26]. In this study, we modified FGF1 silk sericin hydrogel materials (FGF1-SS hydrogel) [27], which can be implanted into the injured site for the treatment of IUA. The material has both physical barrier support and stable and sustained release of FGF1, prolonging the half-life and local concentration of FGF1. It is proved that FGF1-SS hydrogel can promote the proliferation of endometrial stromal cells (ESCs) and inhibit endometrial fibrosis for a long time. The effect of FGF1-SS hydrogel on the restoration of uterine structure and fertility was evaluated.

## Materials and methods

### Fabrication of the FGF1-SS hydrogel materials using the genetically engineered silk fibers

In this study, the FGF1 transgenic silkworm B10 line [28] constructed based on piggyBac's transgenic vector phShFGF1Sv40 was kindly provided by Biological Science Research Center, Chongqing Key Laboratory of Sericultural Science, Chongqing Engineering and Technology Research Center for Novel Silk Materials, Southwest University. Grind the genetically engineered FGF1 transgenic silkworm cocoons, and dissolve the cocoon powder in a 0.5% (w/v) 8 M urea solution to extract sericin. After extraction, the FGF1 content in the sericin aqueous solution was quantified by performing an optical density analysis on FGF1 on a Western blot using FGF1 standards. Then, the sericin and FGF1 were dialyzed with deionized water at 4°C, and the deionized water was replaced at least six times within 3 days to completely remove urea and salt. FGF1-SS hydrogel may be formed within 7 days of continuous dialysis. After the same processing, wild-type silkworm cocoons were also used to make WT-SS hydrogel (WT). The total protein content of both WT-SS hydrogel and FGF1-SS hydrogel (WT+FGF1) was 1.2 mg/ml, and the FGF1-SS hydrogel FGF1 content was 1.1 μg/ml.

### Scanning electron microscopy

The platinum layer of sericin hydrogel freeze-dried using a freeze dryer (Alpha1-2, Martin Christ, Germany) was vacuum-plated (NeoCoater MP-19020NCTR) and placed under a scanning electron microscope (SEM, JSM-5610LV, Japan). Observe the substructure morphology of sericin hydrogel under an accelerated working voltage of 10 kV at room temperature. Use Image Pro Plus (version 6.0.0.260) to calculate the average pore size of the sericin hydrogel from 50 random holes.

### Fourier transform infrared spectroscopy analysis

The sericin hydrogel was frozen in liquid nitrogen for 5 min, and the water in the hydrogel was lyophilized in a lyophilizer to perform a Fourier transform infrared spectroscopy (FTIR) test. A Fourier transform infrared spectrometer was used to evaluate the secondary structure of the dried sericin hydrogel samples. The ZnSe ATR cell was in the spectral range of 4000–650 cm^−1^. Use Omni, PeakFit v4.12 and Origin Pro 8 software to analyze the data, and take the average of the independent deconvolution of at least 30 independent tests for each sample.

### Release of FGF1 from silk fibers and sericin hydrogels

Add 500 μl of FGF1 sericin hydrogel to the wells of a 24-well plate, while adding 500 μl of phosphate-buffered saline (PBS) (pH 7.4) and keep it at 37°C. At each time point, transfer the supernatant of the well to a test tube, and add the same volume of fresh 500 μl PBS (pH 7.4) to the same well. The content of FGF1 in the supernatant was determined by ELISA.

### Viscosity measurement of sericin hydrogel

The viscosity measurement of FGF1 sericin hydrogel was performed on a rheometer with parallel plates (60 mm) in continuous flow mode. The viscosity of FGF1 sericin hydrogel at 25°C at shear rate (1–100 s^−1^) was recorded.

### *In vitro* degradation of FGF1-SS hydrogels

An average of 100 mg of dried sericin hydrogel was incubated in 1 ml PBS (pH 7.4) at 37°C with or without 10 U/ml lysozyme. The PBS was changed daily, and samples were removed at fixed times to dry and weigh. Samples for each time point were made in triplicate.

### *In vivo* experiments on FGF1-SS hydrogels

In order to detect the retention time of FGF1-SS hydrogel in rats, we injected 50 μl of FGF1-SS hydrogel *in situ* in IUA rats, and sacrificed the rats at 1, 6, 12, 24, 48 h, 7 and 14 days after injection. The FGF1 antibody immunohistochemistry was performed on the rat uterus to detect the residence time of FGF1-SS hydrogel in the rat.

### Isolation of rat ESC

Take out the uterus of 9-day gestation rats in a sterile environment and wash them with Hank’s Balanced Salt Solution three times. Cut the uterus into small pieces of 1 mm^2^, add 3 ml of 1 mg/ml type I collagenase (17018029, Gibco, USA), digested at 37°C for 60 min, and stirred every 15 min. Filter the mixed cell suspension through a 40 μm cell strainer, collect the cell suspension and centrifuge at 800 rpm/3 min, add 10% fetal bovine serum DMEM/F-12 medium (01-172-1ACS, BI, ISR), and inoculate it in a 25 cm^2^ culture flask, placed in a 37°C, 5% CO_2_ incubator. After 3 days, observe the cell growth status. When the ESCs were overgrown in the culture flask, the cells were digested with 0.25% trypsin, inoculated on glass slides with 1 × 10^5^, and fixed with 4% paraformaldehyde solution for 30 min, and the expression of vimentin was detected by immunofluorescence.

Hydrogen peroxide (20 μM) (H_2_O_2_, sinopharm, China) was used to damage ESC for 24 h to establish endometrial injury cell model. The WT+FGF1 were co-cultured with H_2_O_2_ injured ESC for 24 h to test the therapeutic effect of the material on the cell injury model.

### IUA rat model

Animal experiments were approved by the National Research Institute for Family Planning Animal Ethics Committee. Female Sprague-Dawley rats aged 7–8 weeks were purchased from Huayikang Biotechnology Co., Ltd to establish an endometrial injury model [29]. The rats were raised in an SPF environment with free water and food. A total of 100 rats were randomly divided into five groups with 20 rats in each group. The five groups were normal group (control), sham group (sham), model group (model), WT treatment group (WT) and WT+FGF1 treatment group (WT+FGF1). The rats in the control group were fed normally; the rats in the sham group underwent surgery and were injected with physiological saline; the rats in the model group were anesthetized by intraperitoneal injection of 3% sodium pentobarbital (0.3 ml/100g), and the lower abdomen was opened to expose the uterine horns. After injecting 0.3 ml of 95% ethanol to injure the uterus for 3 min, the uterus was washed twice with PBS to establish the injury model; in the WT group and the WT+FGF1 group, 50 μl of WT and WT+FGF1 were injected into the unilateral uterus *in situ* after uterine injury, respectively. Record the day of modeling as Day 0. At 30 and 60 days after modeling, five rats from each group were randomly selected to be sacrificed and five rats were mated.

### Determination of endometrial thickness

Endometrial thickness (from the basement membrane to the apical surface of the epithelium) is determined by measuring the distance along a transverse section of the proximal and distal ends of the endometrium, which is visually considered to be the thickest of any single site [30]. All samples were measured, five fields of view were taken for each section, and the average value was recorded.

### Fertility test

On the 30th and 60th day after modeling, five female rats in each group were randomly selected to mate with male rats with proven fertility in a ratio of 1:1. Uterine function is evaluated by detecting the pregnancy ability of the uterus. The morning when the vaginal plug is present is considered to be the 0.5th day of pregnancy. On the ninth day of pregnancy, female mice were sacrificed for uterine examination and the number of fetuses in each uterus was checked.

### Histological and immunohistochemistry analysis

Uterine tissue was fixed overnight with 4% paraformaldehyde, dehydrated by ethanol gradient, and then embedded in paraffin. The uterine tissue was sliced (4 μm), stained with hematoxylin and eosin (H&E), Masson stain and Sirius red stain to observe the structure of the uterus. A TE2000-U inverted microscope (Nikon, Tokyo, Japan) was used to observe the uterine morphology and endometrial thickness. Three High power field randomly selected for each image were averaged, and the number of uterine glands was counted. Image J software for analysis and degree of endometrial fibrosis [31]. The fibrotic area was determined using the following formula, using at least five mice per group:

$$\text{Fibrotic area}\;(\%)=(\frac{\;\mathrm{total}\;\mathrm{area}\;\mathrm{of}\;\mathrm{endometrial}\;\mathrm{fibrosis}\;\mathrm{per}\;\mathrm{field}}{\mathrm{the}\;\mathrm{sum}\;\mathrm{area}\;\mathrm{of}\;\mathrm{endometrial}\;\mathrm{stroma}\;\mathrm{and}\;\mathrm{gland}})\times 100.$$

The type I collagen area was determined using the following formula, using at least five mice per group:

$$\mathrm{Type}\;I\;\mathrm{collagen}\;\mathrm{area}\left(\%\right)=(\frac{\mathrm{total}\;\mathrm{area}\;\mathrm{of}\;\mathrm{endometrial}\;\mathrm{type}\;I\;\mathrm{collagen}\;\mathrm{per}\;\mathrm{region}\;}{\mathrm{the}\;\mathrm{sum}\;\mathrm{area}\;\mathrm{of}\;\mathrm{endometrial}\;\mathrm{stroma}\;\mathrm{and}\;\mathrm{gland}})\times 100.$$

Add 10 mM citrate buffer to the paraffin tissue section to restore the antigen under high pressure, and add the primary antibody at 4°C overnight. The primary antibody used is anti-vimentin (V6630, Sigma-Aldrich, 1:200), anti-α-SMA (ab124964, abcam, 1:200) and anti-FGF1 (SAB1405808, Sigma, 1:200). After washing three times with PBS, add anti-horseradish peroxidase Conjugate anti-rabbit IgG (ZB-2306, ZSGB-BIO, 1:1000) and incubate at room temperature for 1 h. Use DAB to develop color for 3 min, add hematoxylin for counterstaining and dehydration and mount. Image J software is used to analyze and evaluate the stained area of brown staining [31].

### Quantitative real-time polymerase chain reactions

The rat uterine RNA extracted by Trizol (Invitrogen, CA, USA) was reverse-transcribed to cDNA using a reverse transcription kit (Transgen Biotech, Beijing, China). Quantitative real-time polymerase chain reaction (qRT-PCR) was performed using mRNA qPCR detection kit (SYBR Green, Bio-Rad, CA, USA) in CFX96 touch deep well real-time PCR detection system (Bio-Rad, CA, USA). Use each sample in each group to perform three replicate tests. Repeat the experiment at least three times. qRT-PCR primer sequence: TGF-β-Forward, 5′-CGGACTACTACGCCAAAGAAG-3′; TGF-β-Reverse, 5′-TCCCGAATGTCTGACGTATTG-3′; PDGFβ-Forward, 5′-ATGACCCGAGCACATTCTGG-3′; PDGFβ-Reverse, 5′-ACACCTCTGTACGCGTCTTG-3′; FGF1-Forward, 5′-AAGGGCTTTTATACGGCTCG-3′; FGF1-Reverse, 5′-CGCTTACA ACTCCCGTTCTT-3′; SUSD-Forward, 5'-GAAGT GGCCTTAGGGCTTGA-3′; SUSD-Reverse, 5′-TGCTATCAGTGAGTGACCTCC-3′; GAPDH-Forward,5′-GCAAGGATACTGAGAGCAAGAG-3′; GAPDH-Reverse, 5′- GGATGGAATTGTGAGGGAGATG-3′.

### Cell proliferation, migration and infiltration ability detection

Cell Counting Kit-8 (CCK-8) detects the proliferation of ESCs. Inoculate ESC in a 96-well plate at 10^4^/well, co-culture with WT-SS/FGF1-SS hydrogel in a 37°C incubator for 24 h, add 10 μl of CCK-8 solution to each well, and incubate in the incubator for 1 h. A microplate reader (Bioteck, Vermont, USA, Gen 5) was used to measure the absorbance at 450 nm, and the average value of the absorbance at five wells was taken to obtain the cell proliferation rate. The experiment was repeated three times.

ESCs co-cultured with WT-SS/FGF1-SS hydrogel for 24 h were seeded into the Transwell chamber of a 24-well plate at 1 × 10^5^ cells/ml, and cell migration was detected after 12 h of culture. The cell infiltration experiment uses a Transwell chamber covered by Matrigel (#354248, BD Biosciences, NJ, USA), and then inoculates 1 × 10^5^ cells/ml ESC in a 24-well plate Transwell chamber. After 20 h of culture, the cell infiltration was detected. Before observation, the cells were fixed with 4% paraformaldehyde and stained with 0.1% crystal violet. The total number of cells in five fields of view was recorded under a 40-fold objective lens and take the average. The experiment was repeated three times.

### Western blot analysis

Uterine tissue and ESCs protein were extracted using RIPA lysate (Beyotime, Shanghai, China). After 15 min of lysis on ice, 12 000 rpm/10 min, the supernatant was taken to quantify the protein (BCA protein detection kit 23225, Thermo Fisher Scientific, USA). A 30 μg protein sample was added to the loading buffer and denatured at 99°C for 10 min, separated on a 10% SDS-polyacrylamide gel and transferred to a PVDF membrane (Amersham, St Albans, UK). Anti-TGF-βI (ab215715, Abcam, Cambridge, UK; 1:500), anti-Smad2/3 (Abcam, Cambridge, UK; 1:200), anti-α-SMA (Abcam, Cambridge, UK; 1:500), anti-pSmad2 (Abcam, Cambridge, UK; 1:200) and anti-pSmad3 (Abcam, Cambridge, UK; 1:200) in Incubate overnight at 4°C. Then, incubate the membrane with anti-rabbit secondary antibody (ZSJQ-Bio, Beijing, China; 1:2000) at room temperature for 1 h. Protein band was visualized using a chemiluminescence kit (New Cell Molecular Biotechnology Co., Ltd, Suzhou, China). ImageJ is used to quantify the intensity of the bands.

### Statistical analysis

Use SPSS version 20.0 for statistical analysis. Quantitative data are mean ± standard deviation. Significant differences between groups were determined by analysis of variance using GraphPad software version 5. *P *<* *0.05 is considered statistically significant.

## Results

### Preparation and characterization of FGF1-SS hydrogel

**Figure 1.**
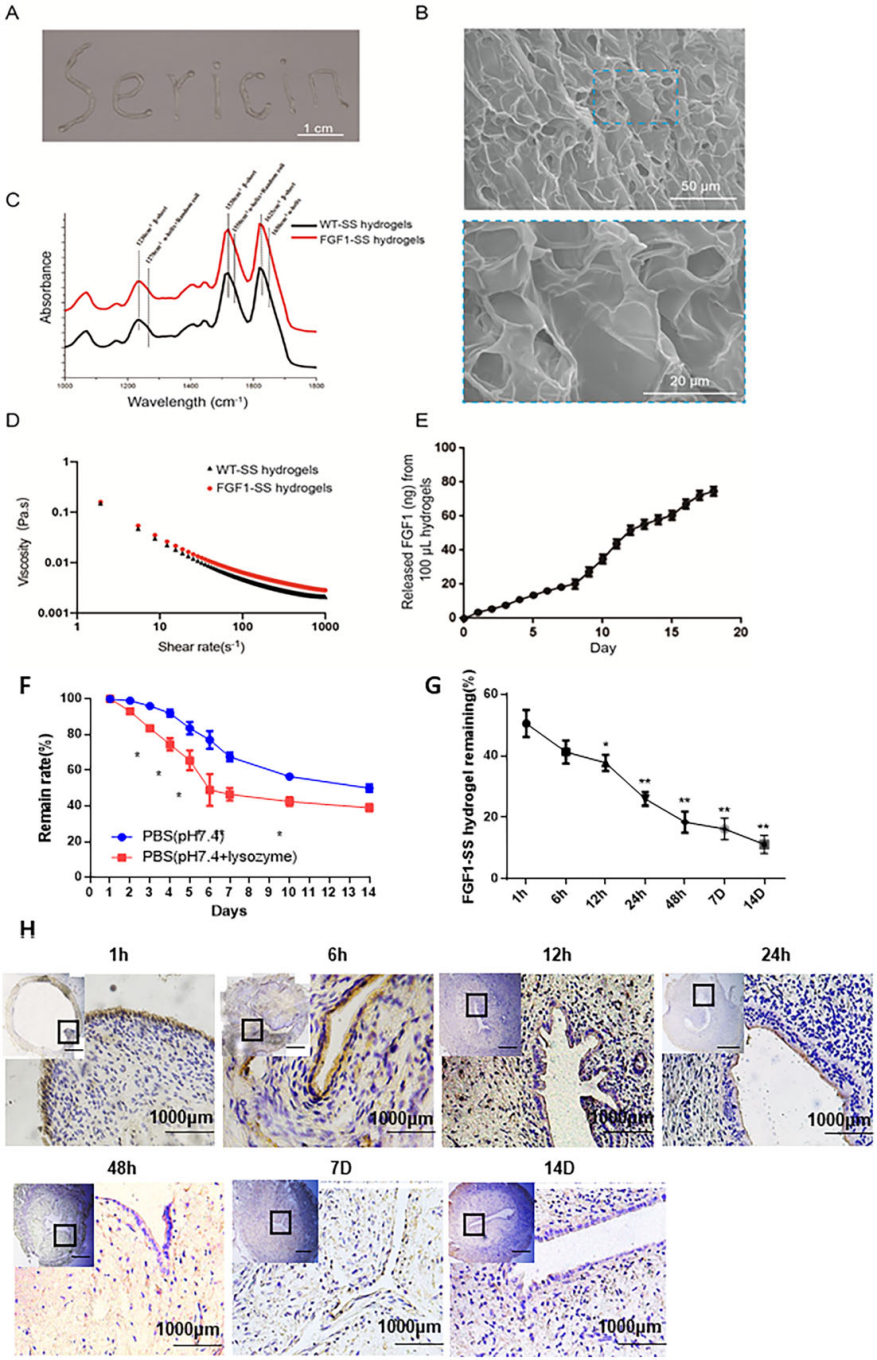
Characteristics of the injectable FGF1-SS hydrogel. (**A**) Digital image of the fabricated injectable FGF1-SS hydrogel, scale bar: 1 cm. (**B**) SEM images of the FGF1-SS hydrogel under at 500-fold magnification (top inset) and 2000-fold magnification (bottom inset), respectively. (**C**) FTIR analysis of the WT+FGF1. (**D**) Viscosity–shear rate relation of the WT+FGF1. (**E**) Release behavior of the FGF1-SS hydrogel. (**F**) The degradation dynamics of the FGF1-SS hydrogel in PBS (pH 7.4) with or without lysozyme. (**G**) Changes of FGF1 expression in rats injected with FGF1-SS hydrogel *in situ*. (**H**) Expression of FGF1 factor in rat uterus to detect the retention behavior of FGF1-SS hydrogels *in vivo*. *P <* *0.05; **P<0.01. *n* = 5

The harvested FGF1 silk powders were dissolved in 8 M urea at a 0.5% (w/v) ratio for the simultaneous extraction of the sericin and the FGF1. The obtained WT-sericin and FGF1-sericin aqueous solution had a total protein concentration of ∼1.20 and ∼1.14 mg/ml, respectively. The content of the FGF1 protein in the FGF1-sericin aqueous solution was further estimated to be 1.10 μg/ml. The FGF1-SS hydrogel formed during the dialysis processing for the removal of the urea and the salts. FGF1-SS hydrogel has an injectable type and can pass through a syringe needle with a minimum size of 0.7 × 35 mm (Fig. 1A). SEM analysis of the FGF1-SS hydrogel showed an interconnected lamellar and porous microstructure morphology (Fig. 1B). Three strong peaks were found in the similar FTIR spectra of WT+FGF1, respectively, which are amide I (1590–1699 cm^−1^), amide II (1480–1570 cm^−1^) and amide III (1200–1310 cm^−1^) protein bands (Fig. 1C). In addition, absorption peaks representing β-sheets were found at 1625, 1530 and 1230 cm^−1^ in both materials, indicating that there is a dense intermolecular hydrogen bond network in the material. Rheological property of the FGF1-SS hydrogel was examined, the results showed the viscosity of the FGF1-SS hydrogel decreased while increasing the shear rate (Fig. 1D), revealing the macroscopically homogeneous and convenient injectability feature of the fabricated material. FGF1-SS hydrogel can continuously and stably release FGF1, and the cumulative release of FGF1 from 100 μl FGF1-SS hydrogel is 78.23 ± 9.37 ng (Fig. 1E).

### *In vivo* experiments on FGF1-SS hydrogels

FGF1 sericin hydrogels degraded rapidly *in vitro* with 48.08% ± 2.47% material loss (in PBS, pH 7.4) for the first 10 days, and then the degradation rate decreased with a total loss of 60.78% ± 1.36% within 14 days (Fig. 1F). In order to detect the retention behavior of FGF1-SS hydrogel *in vivo*, we took the IUA rats injected with FGF1-SS hydrogel *in situ* at 1, 6, 12, 24, 48 h, 7 and 14 days, respectively, after injection. Because FGF1 factor is of human origin in FGF1-SS hydrogel, we could distinguish human FGF1 from rat FGF1 with anti-human FGF1 antibody in rat uterus. It can be seen from the results of immunohistochemistry that FGF1 can enter the uterine cavity epithelium at 1 h, and then migrate to the endometrium. After 24 h, it completely enters the endometrium, and the luminal epithelium has no FGF1 expression. The expression of FGF1 gradually decreased over time (Fig.1G and H). Therefore, we speculate that FGF1-SS hydrogel is not cleared quickly after the uterus, but acts by infiltrating the uterine cavity epithelium and then into the endometrium.

### The effect of FGF1-SS hydrogel on ESCs

**Figure 2.**
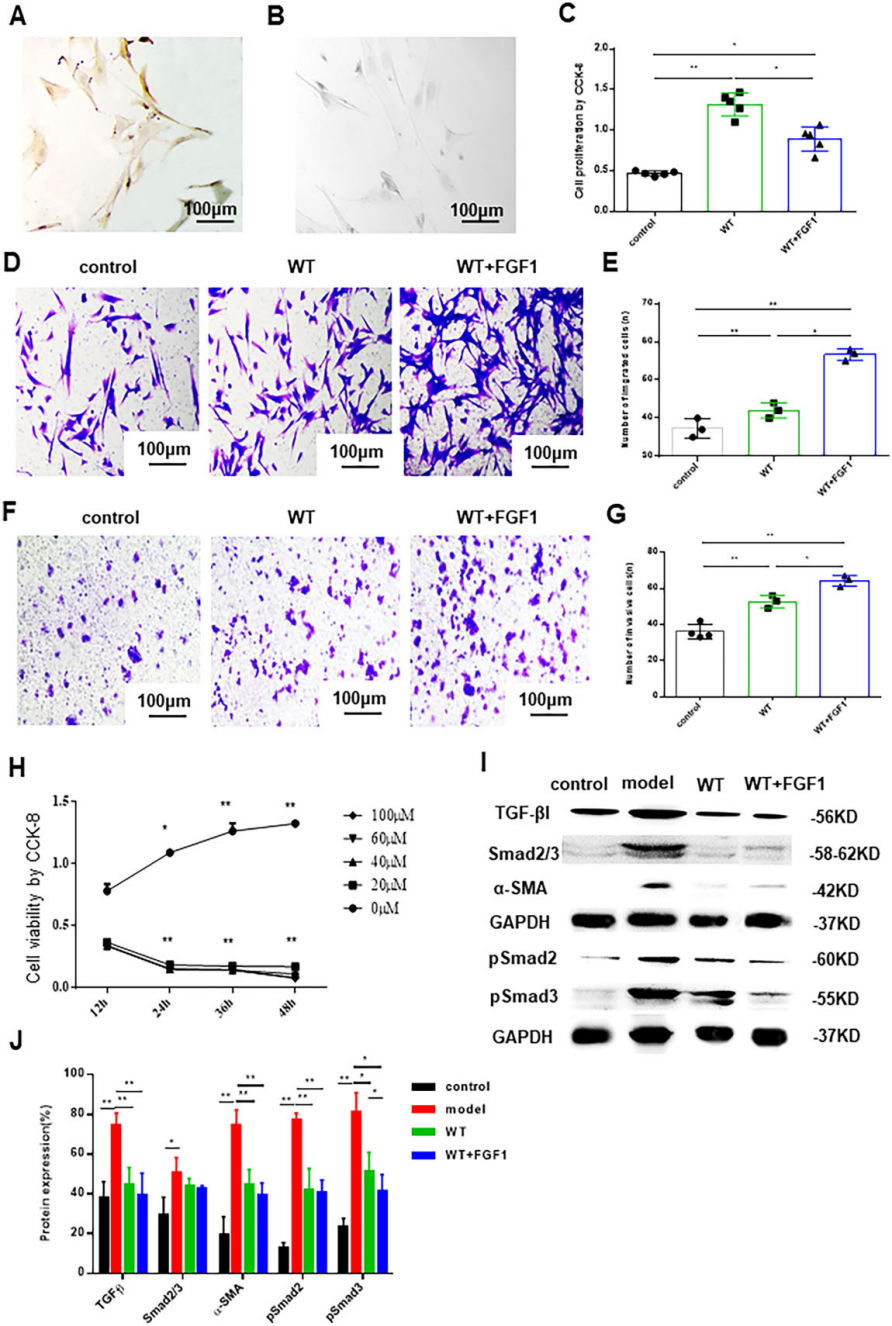
The effect of FGF1-SS hydrogel on ESCs. (**A**) Vimentin immunohistochemical identification of ESCs was positive. (**B**) Identification of ESCs negative by keratin immunohistochemistry. (**C**) CCK-8 detects the cell proliferation effect of FGF1-SS hydrogel on ESCs, *n* = 5. (**D** and **E**) Transwell chamber to detects the cell migration effect of FGF1-SS hydrogel on ESCs. Scale bar: 100 μm, *n* = 3. (**F** and **G**) The cell infiltration effect of FGF1-SS hydrogel on ESCs, scale bar: 100 μm, *n* = 3. (**H**) H_2_O_2_ constructs damage model of ESCs, *n* = 5. (**I** and **J**) Detecting sericin hydrogel and FGF1-SS hydrogel in the endometrial cell injury model to treat fibrosis. *P <* *0.05 and **P <* *0.01

Because the basal layer of the endometrium is damaged and the endometrium cannot be repaired, it is essential to restore the cellular function of ESCs. In order to test the effect of FGF1-SS hydrogel on the cell function of ESCs, we isolated rat ESCs, and immunohistochemical identification of vimentin was positive and keratin was negative (Fig. 2A and B). The FGF1-SS hydrogel was co-cultured with ESCs for 24 h. CCK-8 detection revealed that the cell proliferation rate of the WT+FGF1 group (0.89 ± 0.1) and WT group (1.36 ± 0.1) was significantly higher than that of the control group (0.48 ± 0.04) (*P *<* *0.05, *n* = 4) (Fig. 2C), the cell migration rate and cell infiltration rate of the WT+FGF1 group (54 ± 7, 64 ± 6) were higher than those of the WT group (42 ± 5, 52 ± 2), and both were significantly higher than the control group (37 ± 4, 36 ± 3) (*P *<* *0.05, *n* = 3) (Fig. 2D–G). It is suggested that FGF1-SS hydrogel material can significantly promote the proliferation, migration and infiltration ability of ESCs. Among them, FGF1-SS hydrogel material releases FGF1, which promotes the migration and infiltration ability of ESCs more significantly than WT material. In order to further study the therapeutic effect of FGF1-SS hydrogel on ESCs injury, we selected 20 μM 24 h to construct an ESCs injury model (Fig. 2H). Because fibrosis is the main cause of IUA, we used western blotting to detect the inhibitory effect of FGF1-SS hydrogel on fibrosis in the ESCs injury model. The results showed that the expression of α-SMA in the WT+FGF1 group was significantly down-regulated compared with the model group, and the expression of TGF-β, pSmad2 and pSmad3 was also significantly down-regulated (*P *<* *0.05) (Fig. 2I and J). It is suggested that FGF1-SS hydrogel treatment can reduce the fibrosis of ESCs damage and play a role by inhibiting the TGF-β/Smad signaling pathway.

### FGF1-SS hydrogel improves the appearance and shape of the uterus

**Figure 3.**
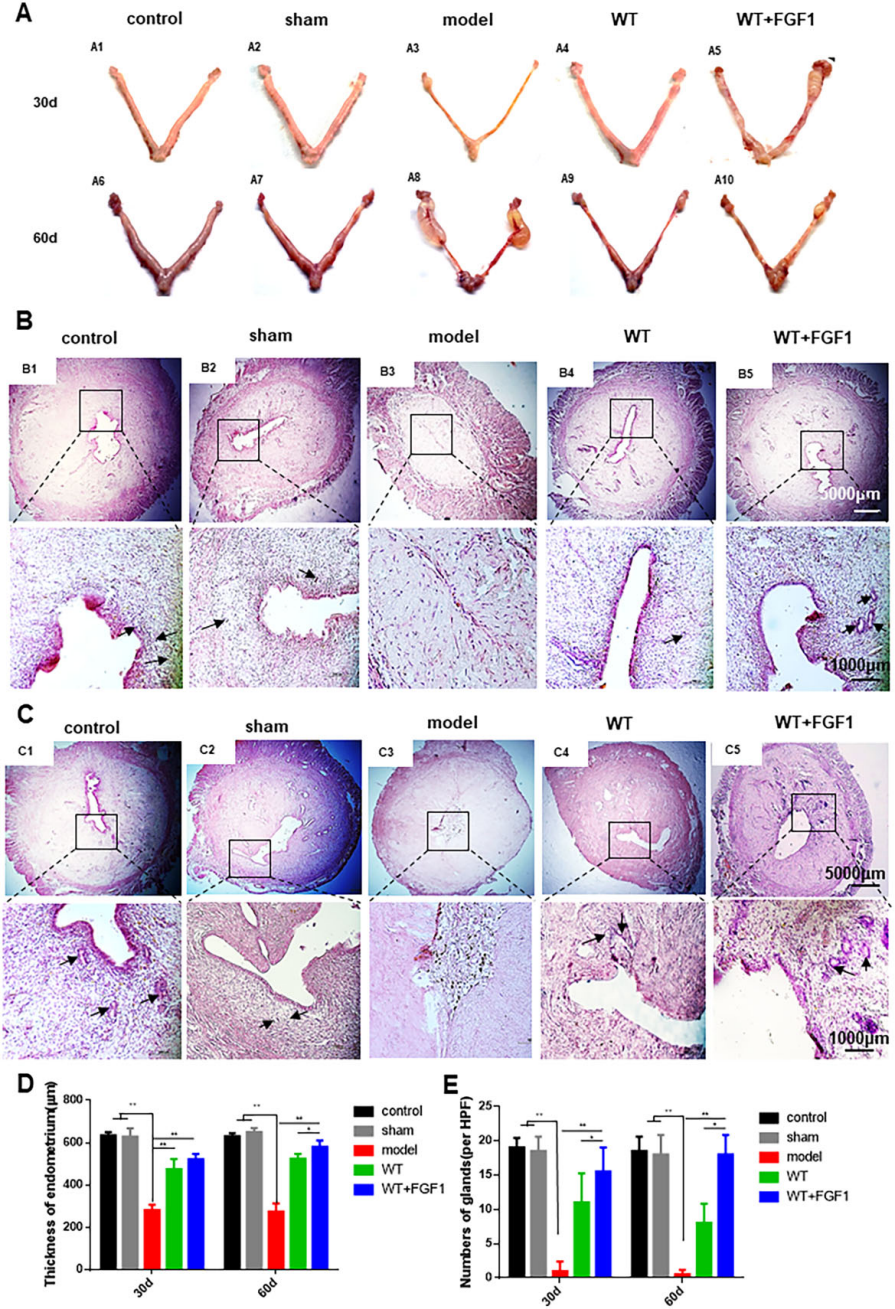
FGF1-SS hydrogel treatment improves rat fertility. (**A**) The appearance of rats in each group was 30 days (A1-5) and 60 days (A6-10) after modeling. (**B**) H&E staining of rat uterine tissue in each group 30 days after modeling, the black arrow points to the uterine gland, scale bar: 1000 μm, *n* = 5. (**C**) H&E staining of uterus in each group 60 days after modeling, scale bar: 1000 μm, *n* = 5. (**D**) Endometrial thickness of each group at 30 and 60 days after modeling. (**E**) The number of uterine glands in each group at 30 and 60 days after modeling. *P <* *0.05 and **P <* *0.01

The uterus of the rats in the control group and the sham operation group was smooth and tough. The uterus in the model group was significantly atrophy, loss of elasticity and edema. Compared with the model group, the uterus in the WT+FGF1 group showed a relatively smooth and plump surface at 30 and 60 days after modeling (Fig. 3A). The uterine structure was observed by H&E staining (Fig. 3B–E). In the model group, the uterine cavity structure disappeared after modeling, and the endometrial thickness at 30 and 60 days after modeling was 356 ± 21 and 339 ± 30, respectively, which was significantly lower than that in the control group (674 ± 55, 681 ± 40, *n* = 5, *P *<* *0.05). At the same time, the number of glands in the model group (0.5 ± 1, 2.67 ± 1, *n* = 5) was significantly lower than that in the control group (19 ± 2, 19 ± 1, *n* = 5, *P *<* *0.05). The WT group maintained a complete uterine cavity structure, and the endometrial thickness (478 ± 71, 467 ± 35, *n* = 5) and the number of glands (12 ± 2, 9 ± 1, *n* = 5) were significantly increased compared with the model group. After 30 days of modeling, the structure of the uterine cavity in the WT group was basically intact, and the cells in the functional layer were neatly arranged. However, the cells in the functional layer of the uterus in the WT group were disordered and the number of glands was significantly lower than that in the WT+FGF1 group after 60 days of modeling. In the WT+FGF1 group, the structure of the uterine cavity was complete 30 and 60 days after modeling, the structure of the endometrium was relatively complete, the thickness of the endometrium (526 ± 40, 570 ± 22, *n* = 5) and the number of glands (14 ± 1, 15 ± 1, *n* = 5) was significantly up-regulated compared with the model group (*P *<* *0.05). Among them, 60 days after modeling, the number of uterine glands and endometrial thickness in the WT+FGF1 group were significantly up-regulated compared with the WT group, suggesting that FGF1-SS hydrogel has a better therapeutic effect on endometrial injury.

### FGF1-SS hydrogel treatment improves rat fertility

**Figure 4.**
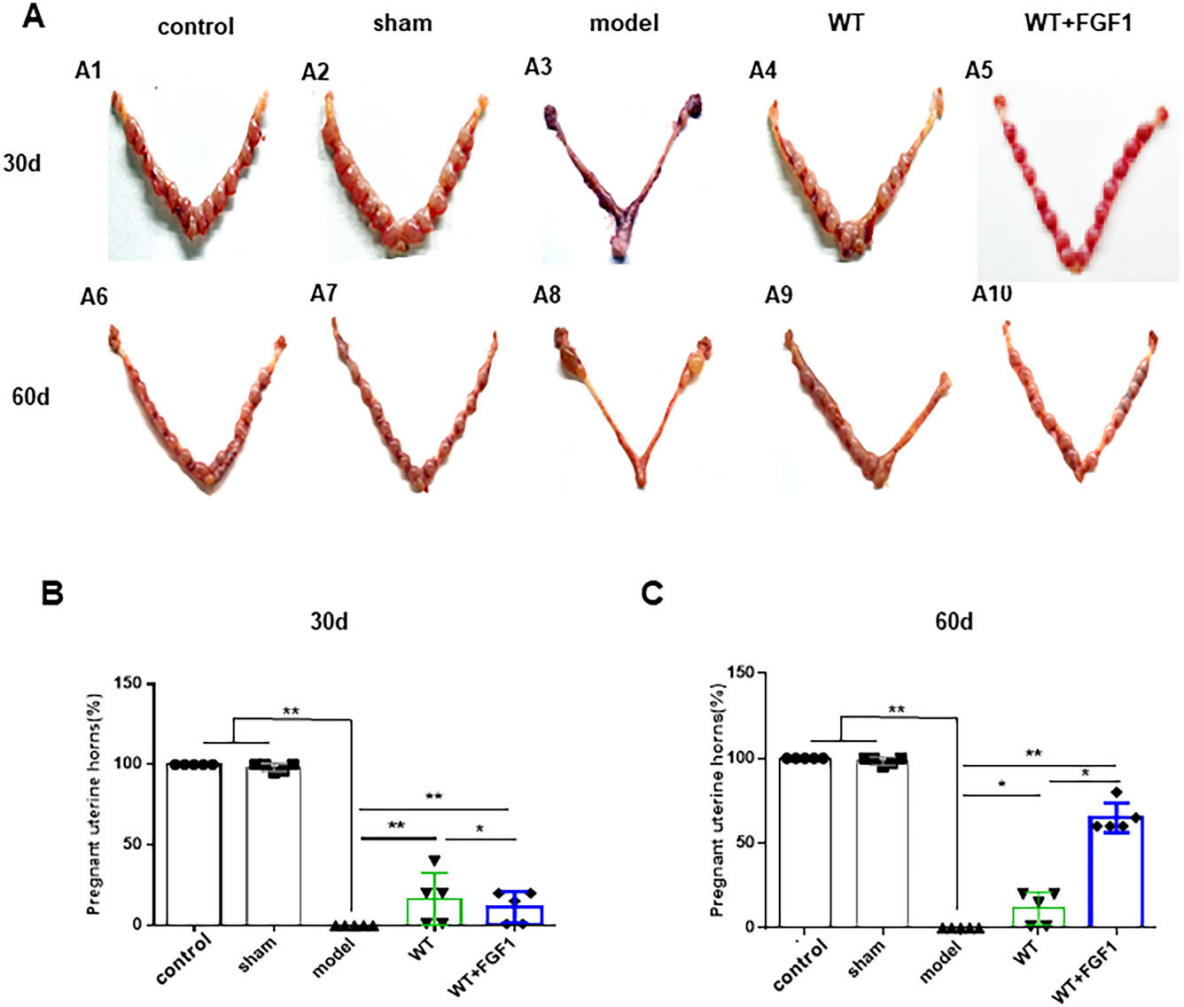
FGF1-SS hydrogel treatment improves rat fertility. (**A**) At 30 days (A1-5) and 60 days (A6-10) after modeling, the appearance of the uterus of rats in each group was seen 9 days after embolization. Statistical analysis of pregnancy rates at 30 days (**B**) and 60 days (**C**) after modeling. *P <* *0.05 and **P <* *0.01

IUA can cause endometrial fibrosis, thereby reducing the rate of embryo implantation. We measure the recovery of uterine function by calculating the implantation rate of rat embryos. As shown in Fig. 4A, 30 days after modeling, the embryo implantation rate in the WT group (40% ± 5%, *n* = 5) was significantly higher than that in the WT+FGF1 group (18% ± 1%, *n* = 5) (Fig. 4B). On the 60th day after modeling, the embryo implantation rate in the WT+FGF1 group (65.1% ± 6.4%, *n* = 5) was significantly higher than that in the WT group (18% ± 1%, *n* = 5) (Fig. 4C). The results show that FGF1-SS hydrogel can restore the uterine injury in IUA rats and maintain the therapeutic effect.

### FGF1-SS hydrogel inhibits endometrial fibrosis

**Figure 5.**
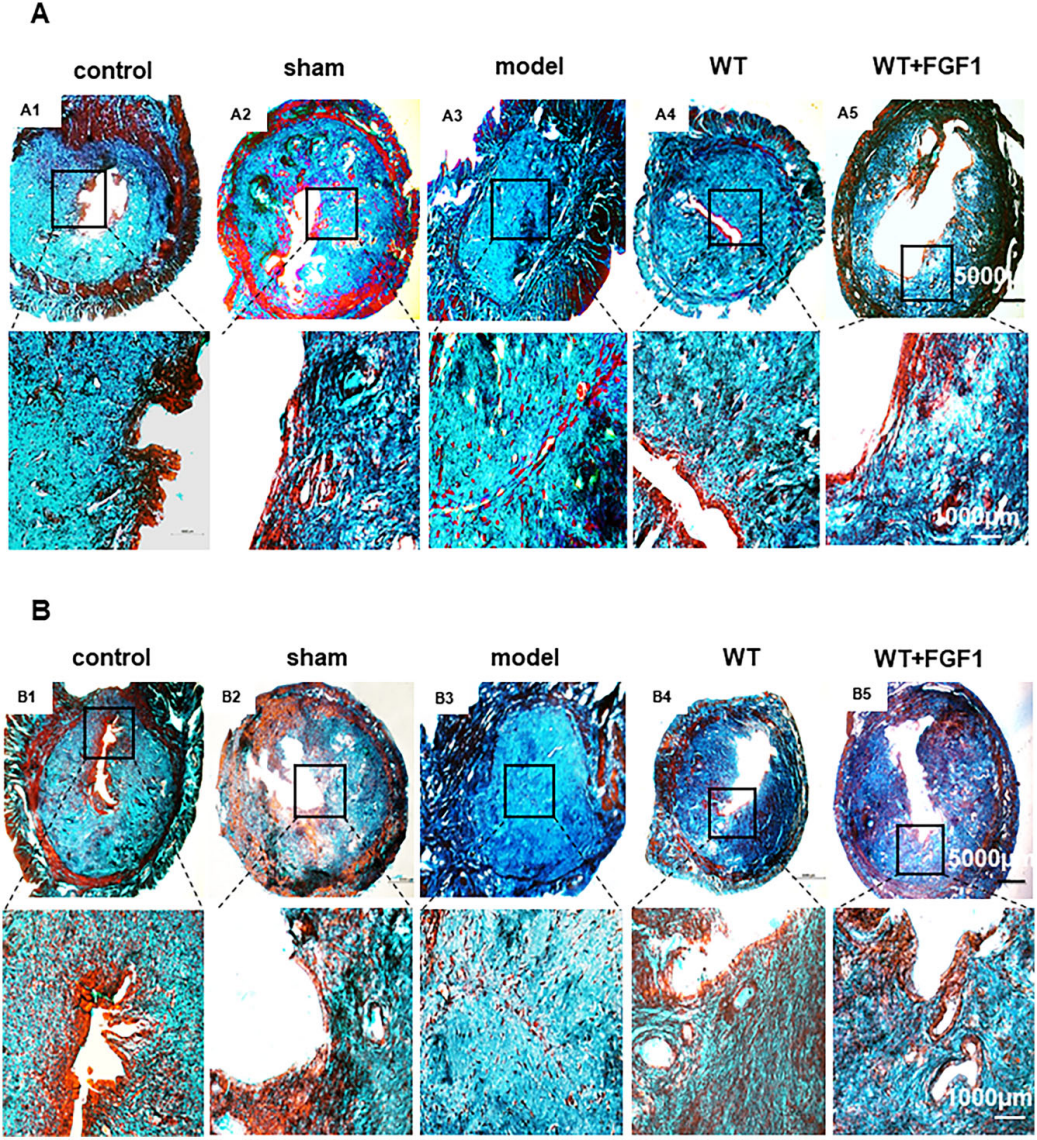
FGF1-SS hydrogel inhibits endometrial fibrosis. (**A**, **B** and **E**) Masson staining to detect uterine fibrosis on the 30th and 60th day. (**C**, **D** and **F**) Sirius red staining to detect type I collagen on the 30th and 60th day. Scale bar = 5000 μm, scale bar = 1000 μm. *n* = 5. *P  <0.05, **P <0.01

A large amount of collagen deposition can lead to fibrosis. To evaluate the inhibitory effect of FGF1-SS hydrogel on fibrosis, we used Masson staining (Fig. 5A, B and E) and Sirius red staining (Fig. 5C, D and F) to analyze collagen deposition in the uterus. The results of Masson staining showed that the degree of fibrosis in the model group (73% ± 5%, 74% ± 3%, *n* = 5) was significantly higher than that in the control group (41% ± 4%, 40% ± 6%, *n* = 5). The degree of fibrosis in the WT group (51% ± 3%, 53% ± 8%, *n* = 5) and the WT+FGF1 group (52% ± 2%, 46% ± 5%, *n* = 5) was significantly lower than that in the model group (*P* < 0.05). At 60 days after modeling, the degree of fibrosis in the WT+FGF1 group was significantly lower than that in the WT group (*P *<* *0.05), suggesting that FGF-SS hydrogel has a long-term therapeutic effect. Sirius red staining was used to detect type I collagen, and the results showed that the WT group (40% ± 1.3%, 45.72% ± 2%, *n* = 5) and the WT+FGF1 group (43% ± 1%, 37% ± 5%, *n* = 5) had type I collagen. The deposition was significantly lower than that in the model group (77% ± 2%, 87% ± 2%, *n* = 5, *P *<* *0.05). The results show that FGF1-SS hydrogel can significantly inhibit uterine fibrosis 30 and 60 days after modeling, and the effect is significantly better than WT.

### The inhibitory effect of FGF1-SS hydrogel on fibrosis

**Figure 6.**
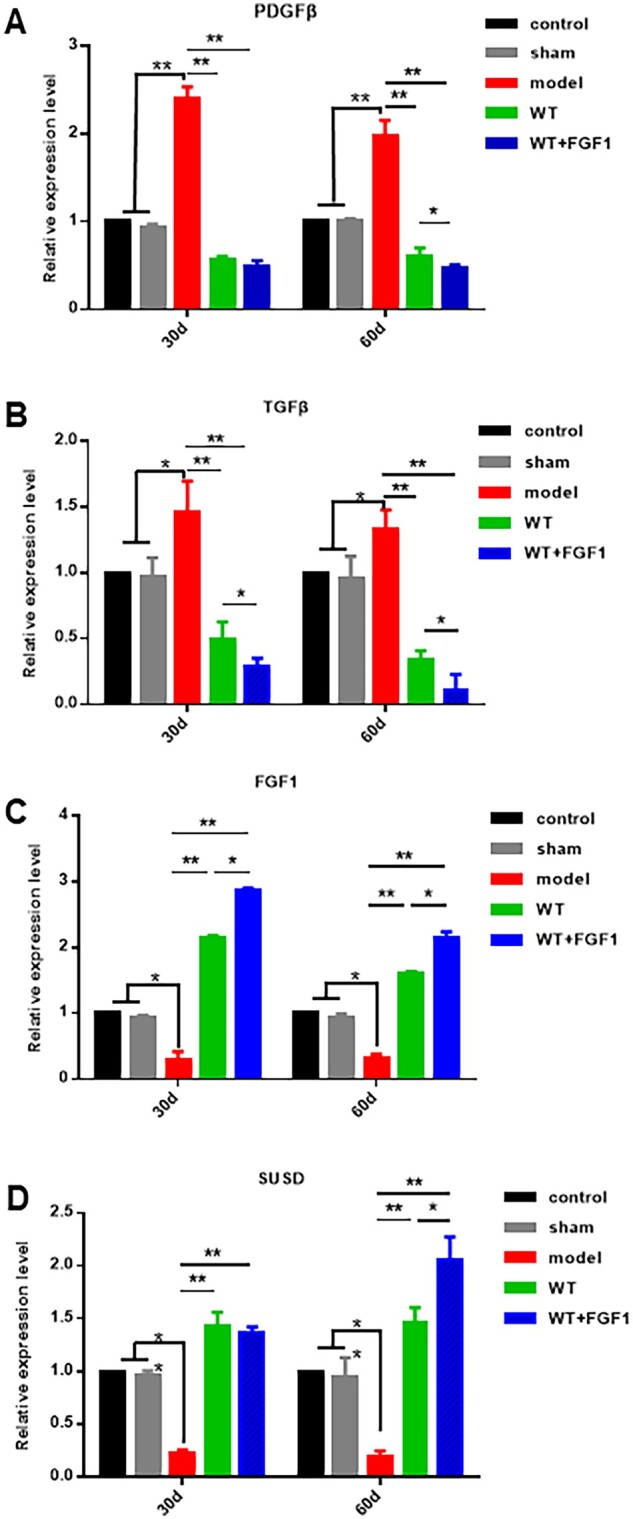
FGF1-SS hydrogel is a related factor in the treatment of IUA. (**A–D**) qRT-PCR to detect the expression of fibrosis-related factors. The expression of TGF-β, PDGFβ, FGF1 and SUSD in rat uterus 30 and 60 days after modeling were detected by qRT-PCR. *P <* *0.05; **P *<* 0.01. *n* = 5

**Figure 7.**
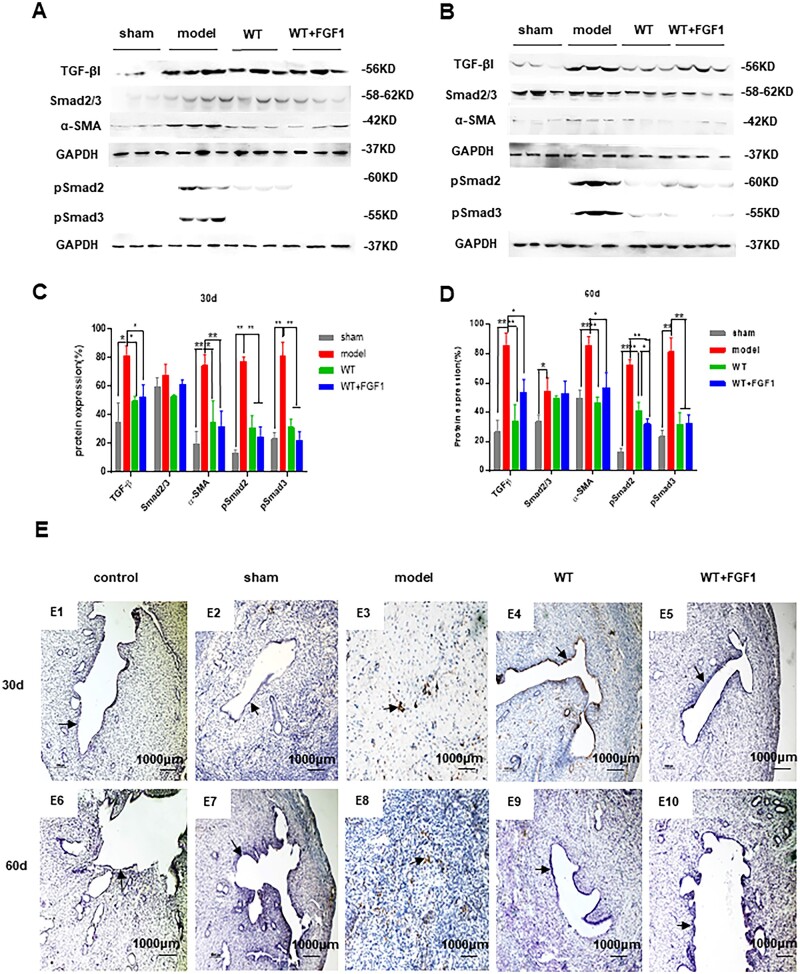
FGF1-SS hydrogel inhibits the expression of fibrosis-related factors. (**A** and **C**) Detect the expression of rat uterine fibrosis-related proteins 30 days after modeling. (**B** and **D**) The expression of rat uterine fibrosis-related proteins 60 days after modeling was detected. (**E**) α-SMA immunohistochemistry to detect endometrial fibrosis

As shown in Fig. 6A–D, the expression of PDGFβ and TGF-β that promote fibrosis in the WT+FGF1 group was significantly down-regulated compared with the model group at 30 and 60 days after modeling (*P *<* *0.05). The expression of FGF1 that inhibits fibrosis and the endometrial stem cell marker SUSD were significantly up-regulated (*P *<* *0.05). The results showed that FGF1-SS hydrogel inhibited the expression of uterine fibrosis factor. Western blot results showed (Fig. 7A–D) the expression of TGF-β1, pSmad2, pSmad3 and α-SMA in the model group was up-regulated compared with the WT group and WT+FGF1 group. The results indicate that FGF1-SS hydrogel may inhibit the expression of pSmad2 and pSmad3 by inhibiting the expression of TGF-β, thereby inhibiting uterine fibrosis. The results of immunohistochemistry showed (Fig. 7E) that the endometrium of the model group was brown, suggesting a large amount of α-SMA expression and endometrial fibrosis. However, the endometrium of the WT+FGF1 group has no obvious positive staining of α-SMA, which indicates that FGF1-SS hydrogel inhibits the formation of endometrial fibrosis and has a good therapeutic effect on IUA.

## Discussion

Bombyx mori silk is mainly composed by the fibroin and sericin, the fibroin accounts for about 75% weight of the cocoon silk, while the sericin accounts the other 25% part [32]. Although the majority part of the silk, fibroin has been used as main textile materials for thousands of years or as newly desirable biomaterials for applications of many aspects of tissue engineering, the sericin once regarded as residual products during the silk degumming processing [33], was recently found to be as a potential biomaterial for biological applications due to its biocompatibility, UV protective property, antibacterial activity, antioxidant, anti-tyrosinase activity, coagulant features and moisturizing capabilities [34–37]. In addition, because the sericin is hydrophilic and distributes in the outer layer of silk, it is easier and more convenient to dissolve the sericin than the fibroin dose to make the sericin related biomaterials. Therefore, in this study, we mainly focused on the strategy to functionalize the sericin to expand its applications in biomedicine fields. Normal uterine function is the foundation of women’s reproductive health. Maintaining the normal physiological structure of the endometrium and recovering it after injury is the key to maintaining uterine function. Uterine fibrosis is usually a complication encountered after uterine surgery, including surgery on the uterine cavity during childbirth, and negative pressure suction, forceps and curettage, mid-term labor induction and incomplete abortion evacuation during pregnancy termination, etc [38–40]. Surgical operations on the uterine cavity can cause damage to the basal layer of the endometrium, scarring leads to the formation of fibrous bridges between the opposite surfaces of the uterine cavity, increasing the risk of uterine fibrosis and even completely eliminating the uterine cavity and causing amenorrhea [41–43]. Uterine adhesions are treated through surgical hysteroscopy to remove the adhesions, and this method has a higher risk of recurrence [44]. Studies have shown that the incidence of re-adhesion of the uterine cavity after surgery is 3.1–23.5%, and the recurrence rate of severe IUA is as high as 62.5% [45]. Therefore, how to effectively prevent postoperative uterine cavity re-adhesion is the key and difficult point of IUA treatment.

Recent studies have shown that sericin materials can promote wound healing [46]. In the wound, the cells lose contact and the production of growth factors and cytokines occurs [47]. Fibroblast migration regulates cell proliferation and collagen regeneration and is a key step in cell repair [48], while sericin increases the number of fibroblasts entering the damaged area [49]. At present, it is believed that the main cause of endometrial fibrosis is the abnormal migration and proliferation of uterine epithelial cells and stromal cells, resulting in abnormal secretion of extracellular matrix proteins and cytokines, leading to the deposition of type I collagen in the endometrium to form endometrial fibrosis [50]. Therefore, the repair of endometrial injury includes endometrial epithelial regeneration, blood vessel and gland repair and growth factor stimulation. This study used 95% to construct a rat endometrial injury model. The materials were taken 30 days and 60 days after modeling, and the uterine structure was observed by H&E staining. The results showed that the uterine cavity of the rats in the model group was completely atretic and could not repair itself, suggesting that we successfully constructed an endometrial injury model. In the WT+FGF1 group, the uterine cavity structure is complete, and the endometrial epithelial cells are arranged neatly and the structure is basically complete. The number of uterine glands and the thickness of the uterus are also significantly restored compared with the model group. FGF1-SS hydrogel for the treatment of endometrial injury has the property of rapidly swelling in water without dissolving, and releasing FGF1 factor [15]. In the uterus, physical support occurs to prevent adhesions, maintain FGF1 factor, and treat the basal layer of the uterus for a long time. It can be seen from the results of immunohistochemistry that FGF1 can enter the uterine cavity epithelium at 1 h, and then migrate to the endometrium. After 24 h, it completely enters the endometrium, and the luminal epithelium has no FGF1 expression. The expression of FGF1 gradually decreased over time. From the data of endometrial thickness and number of glands and the degree of fibrosis, it can be seen that WT itself had the same or even better repair effect than FGF1-SS hydrogel on the damaged endometrium on Day 30 and had worse repair effect than FGF1-SS hydrogel on Day 60. It can be seen that WT itself has an effect on the repair of endometrial damage, but its sustainable time is short, and the addition of FGF1 can significantly prolong its repair effect. Therefore, in the short term, WT+FGF1 may have the same or even better repair effect, but in the long term, the repair effect of FGF1 is better. In the fertility test, the uterine fertility is restored. It suggests that FGF1-SS hydrogel have a significant recovery effect on the treatment of endometrial injury in rats. Masson staining showed that the area of uterine fibrosis in the WT+FGF1 group was significantly lower than that in the model group, indicating that FGF1-SS hydrogel have a therapeutic effect on endometrial injury. In Sirius red staining, it was found that the accumulation of type I collagen in the model group was significantly up-regulated compared with the WT+FGF1 groups, indicating that FGF1-SS hydrogel inhibited the accumulation of type I collagen in the uterus and inhibited endometrial fibrosis.

Among the many hypotheses about the cause of endometrial fibrosis, MET [51] and endometrial stem cell regeneration and differentiation have been studied. In the IUA clinical study of Zhou *et al.* [52], it was found that estrogen therapy significantly inhibited the expression of TGF-β in the uterus and inhibited the occurrence of fibrosis; studies, such as Se-Ra, found that stimulating endometrial stem cells can restore the differentiation and migration of endometrial cells [53]. Therefore, in this study, the expression of endometrial stem cell marker SUSD and myofibroblast protein marker α-SMA were selected to detect the ways of FGF1-SS hydrogel to inhibit uterine fibrosis and their therapeutic effects on uterine fibrosis. The results showed that compared with the model group, the expression of endometrial marker SUSD in the WT-FGF1 group was significantly up-regulated, and the expression of TGF-β was significantly down-regulated. By detecting the TGF-β/Smad signaling pathway, it was found that FGF1-SS hydrogel inhibited the expression of α-SMA by inhibiting the expression of pSmad2 and pSmad3. We used qRT-PCR and Western blotting to detect signaling pathways related to uterine fibrosis. In qRT-PCR detection, we found that the expression of PDGFβ and TGF-β in the WT+FGF1 group was significantly lower than that in the model group. Studies have shown that PDGFβ and TGF-β are related to organ fibrosis, showing that treatment with sericin materials can slow down the occurrence of uterine fibrosis. Western blot results show that sericin hydrogel and FGF1-SS hydrogel inhibit the expression of α-SMA by inhibiting the TGF-β/Smad pathway, thereby reducing fibrin deposition on the wound surface.

Sericin has natural cell adhesion, which can support cell adhesion and long-term cell growth on its surface or in the scaffold [54], so it can be seen that the cell proliferation rate in the WT group and the WT+FGF1 group is significantly up-regulated compared to the control group. ESCs may undergo epithelial cell transformation under the induction of FGF1 [55, 56], so the cell proliferation rate is lower than that of the WT group. At the same time, FGF1 enhanced chemokines and significantly promoted the migration and infiltration capacity of ESCs (Fig. 2D–G). ESC oxidative damage model *in vitro* was constructed to detect the therapeutic effects of FGF1-SS hydrogel on uterine functional layer damage. The results showed that in the ESC oxidative damage model, FGF1-SS hydrogel inhibited the occurrence of fibrosis by inhibiting the TGF-β/Smad pathway. In cell function experiments, it was found that FGF1-SS hydrogel significantly up-regulated the cell proliferation, migration and infiltration capacity of ESC, suggesting that FGF1-SS hydrogel may treat oxidative damage by changing the function of ESC cells.

The results show that FGF1-SS hydrogel has a significant therapeutic effect on rat endometrial injury and can restore uterine function. It is suggested that FGF1-SS hydrogel can be used as an auxiliary material after uterine cavity surgery to prevent endometrial fibrosis.

## Conclusion

FGF1-SS hydrogel inhibit uterine fibrosis by inhibiting the TGFβ/Smad pathway and block the process of endometrial fibrosis in rats, thereby restoring uterine function. FGF1-SS hydrogel has a long-term therapeutic effect on endometrial injury, and can be used as an auxiliary material for IUA postoperative treatment.

## Ethics approval

The animal-related experiments, including the isolation of human umbilical cord mesenchymal stem cells and the mouse premature ovarian failure modeling, were approved by the China of National Research Institute for Family Planning (Ethics Number 2011-10). All applicable institutional and national guidelines for the care and use of animals were followed.

## Authors' contributions

C.-Y.G. wrote the manuscript, F.W. production materials, X.-C.S. and L.Z. performed animal experiments and data processing, D.Z. and H.W. performed molecular experiments, Q.-Y.X., X.M. and H.-F.X. provided funding and experimental guidance.

## Funding 

This work was funded by grants from the National Key Research and Development Program of China (2016YFC1000803), the National Natural Science Foundation of China (No. 32030103).

*Conflict of interest statement*. The authors declare no competing financial interest.

## Data availability

The datasets during and/or analyzed during this study available from the corresponding author on reasonable request.
